# Sequential development of non-arteritic anterior ischemic optic neuropathy in a patient on hemodialysis

**DOI:** 10.3205/oc000073

**Published:** 2017-09-01

**Authors:** Mukesh Jain, Renuka Srinivasan, K. Ramesh Babu, M. Swapnil Parchand

**Affiliations:** 1 Department of Ophthalmology, JIPMER, Dhanvantri Nagar, Gorimedu, Puducherry, India

## Abstract

**Purpose:** Non-arteritic anterior ischemic optic neuropathy (NA-AION) is typically a disorder of patients 50 years and older predisposed to the vascular and structural optic disc risk factors. We present an interesting case of sequential development of NA-AION in a 45-year-old patient with end-stage renal disease undergoing hemodialysis.

**Methods:** Observational case report.

**Results:** A 45-year-old female on hemodialysis for chronic renal failure complained of sequential acute onset sudden painless gross diminution of vision in right eye followed by the left eye. At presentation, fundus examination revealed secondary optic atrophy and a pallid disc edema with few hemorrhage in the right and left eye, respectively. Ambulatory blood pressure recorded nocturnal dips of diastolic blood pressure. Fundus fluorescein angiography showed hypoperfusion of the left optic disc, confirming the diagnosis of NA-AION. She was treated with oral steroids, but with no improvement.

**Conclusion:** Both treating nephrologist and ophthalmologist should be aware of this uncommon but potentially blinding complication, to permit its early recognition and prevent the occurrence in the fellow eye. Also, care should be taken to prevent and treat any hypotensive episodes during and following dialysis therapy in such high-risk patients.

## Introduction

Non-arteritic anterior ischemic optic neuropathy (NA-AION) is the most common cause of acute optic neuropathy in older age. It is caused due to circulatory insufficiency within the optic nerve head [1], [2] The common predisposing factors associated with NA-AION are systemic hypertension, diabetes, and hypercholesterolemia. A small crowded disc is seen in a majority of patients with NA-AION. The relatively inflexible lamina cribrosa provides the setting for a compartment syndrome within the optic nerve head, resulting in a vicious cycle of ischemia and compression. According to Newman et al., the chance of the fellow eye being involved is 15% at five years [3]. It is rarely seen in patients below 50 years of age. Few case reports have documented occurrence of NA-AION in individuals below 50 years of age with hyperhomocysteinemia, thrombophilia (lupus anticoagulants, anticardiolipin antibodies, prothrombotic polymorphisms (factor V Leiden), and deficiencies of protein C, S, and antithrombin III), severe haemorrhage with secondary hypotension, anemia and in patients on dialysis for chronic renal failure [4], [5], [6], [7]. Arnold et al. showed that the fellow eye involvement rate was significantly higher in young patients compared to the elderly; 42.6% versus 29.6% [6]. We report a rare case of sequential development of NA-AION in a patient with end-stage renal disease undergoing hemodialysis.

## Case description

A 45-year-old female complained of gross diminution of vision in the left eye (LE) on awakening 20 days before. She had a similar history of sudden onset painless diminution of vision in the right eye (RE) two months before. She had diabetes, hypertension, and anemia with chronic kidney disease. She was on hemodialysis bi-weekly for the last 3 years. She gave a history of dialysis a day prior to both of the episodes of acute visual loss. She had no history of headache, pain on extra-ocular movements, limb-weakness, transient ischemic attacks, fever, myalgia, jaw claudication, seizures or focal neurological deficit.

**Figure 1:**
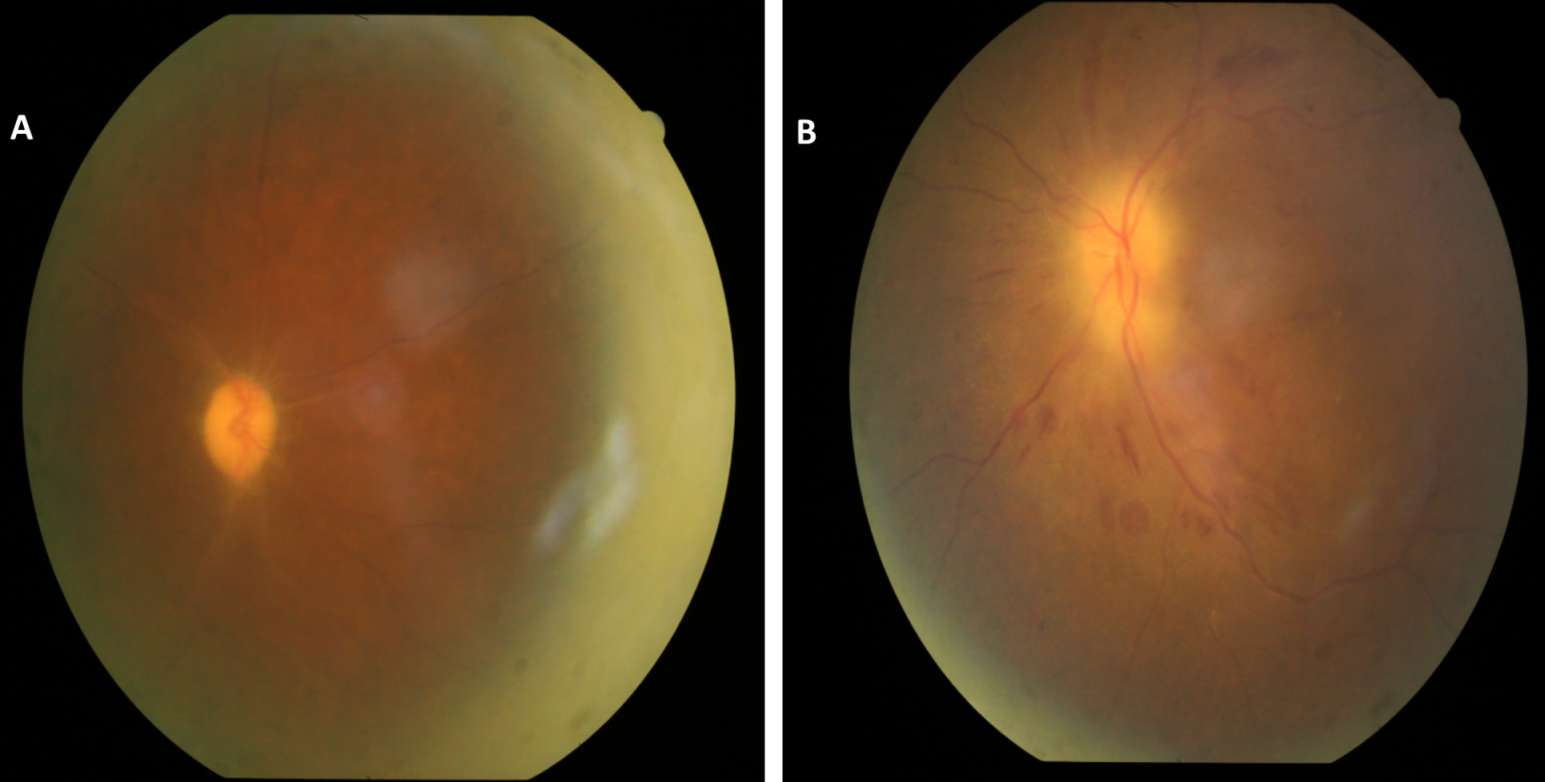
Fundus picture of RE showing optic disc pallor with arteriolar attenuation (A). Fundus picture of LE showing pallid disc edema, flame-shaped retinal hemorrhages in peri-papillary region and attenuation of retinal arterioles (B).

On examination, best-corrected visual acuity (BCVA) was 6/60 in RE and hand movements close to face (HMCF) with accurate projection of rays in LE. Intraocular pressure was 14 and 15 mm of Hg in RE and LE, respectively. LE had grade 1 relative afferent pupillary defect (RAPD). Anterior segment examination of both eyes (BE) was normal. Fundus examination in RE showed optic disc pallor with obliteration of cup and sheathing of vessels around the disc suggesting secondary optic atrophy (Figure 1A (Fig. 1)) with arteriolar attenuation and arterio-venous (A-V) crossing changes. Fundus examination of LE showed pallid disc edema with flame-shaped retinal haemorrhages in the peri-papillary region, attenuation of the retinal arterioles, and arterio-venous crossing changes without any background diabetic retinopathy (Figure 1B (Fig. 1)).

**Figure 2:**
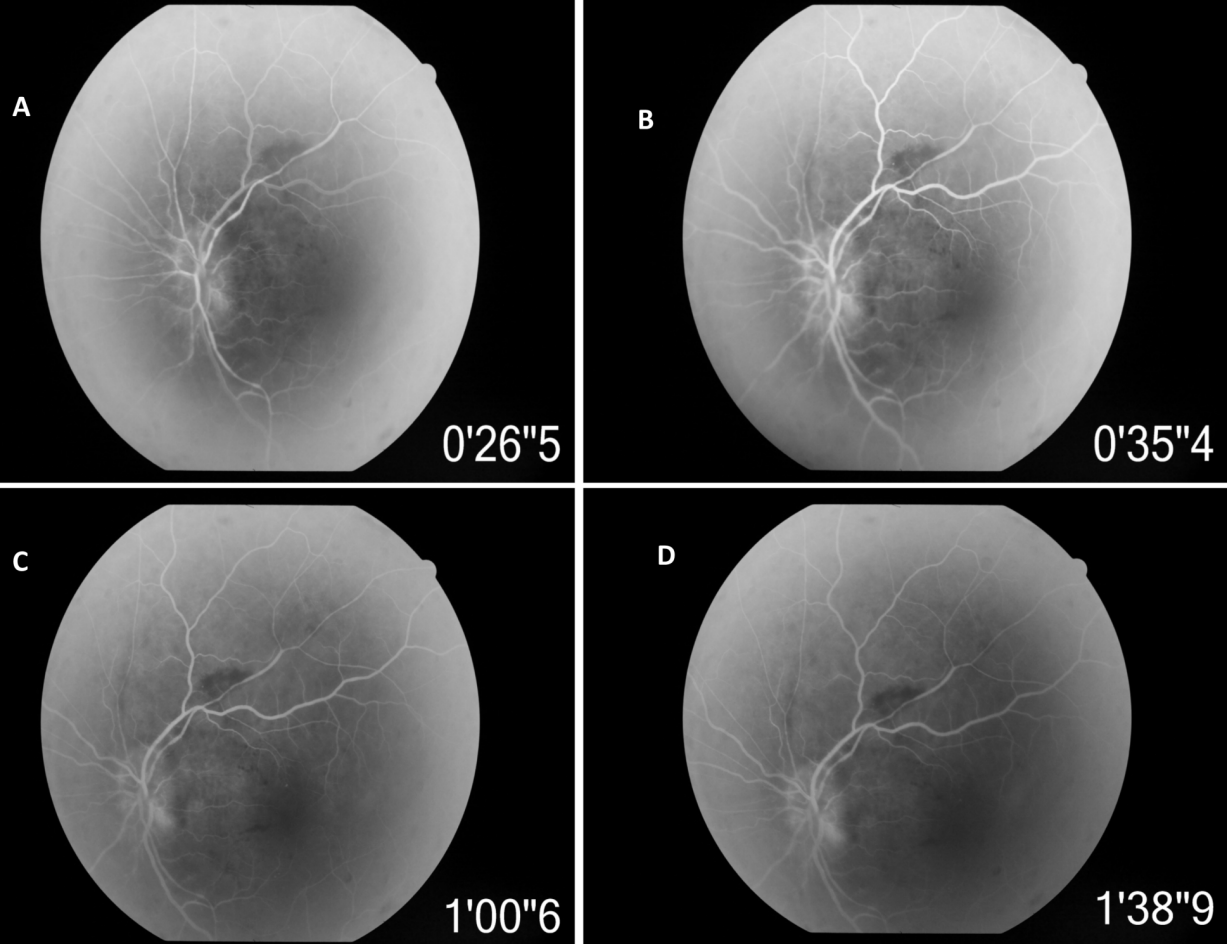
Fundus fluorescein angiography (FFA) of LE showing delayed filling of the disc especially superior part in the early phase, irregular filling of retinal arterioles and staining of the disc in the late phase.

Humphery’s visual field testing in RE showed severely depressed field. Field examination in LE was not possible due to profound visual loss. Fundus fluorescein angiography (FFA) of LE showed delayed filling of the disc especially superior part in the early phase and staining of disc in the late phase (Figure 2 (Fig. 2)). 

Her blood pressure was 110/80 mmHg. Ambulatory blood pressure monitoring showed a drop in nocturnal blood pressure with diastolic reading dipping to 55 mmHg. There was no evidence of any neurological deficit. Her haemoglobin level was 9.5 g/dl. Her blood urea was 78 mg/dl and serum creatinine was 6.2 mg/dl. Serum electrolytes and other routine biochemical tests were within the normal range. Contrast enhanced computer tomography of the head revealed normal study. 

Based on the above findings, she was diagnosed as having secondary optic atrophy following NA-AION in RE and fresh NA-AION in LE. She was started on oral steroid 80 mg/day with close monitoring of blood sugar levels and blood pressure. Steroids were tapered over 8 weeks. Despite these measures, there was no improvement of vision in LE. At last follow-up at 6 months, best corrected visual acuity was 6/60 in RE and HMCF in LE with BE showing secondary optic atrophy.

## Discussion

NA-AION is a disease of the elderly presenting typically with sudden onset painless diminution of vision, mostly reported on awakening [1], [2]. Clinical features include RAPD and disc edema. Optic Disc edema can be diffuse or segmental. Most common documented visual field defect in NA-AION is the altitudinal defect [2]. But in many of these cases, visual field may be severely depressed or non-recordable [8]. FFA helps to confirm the diagnosis by demonstrating delayed disc filling suggestive of hypo-perfusion and resulting ischemia. Insufficiency of optic disc circulation exacerbated by structural crowding of nerve fibers and supporting structures at the nerve head leads to inadequate oxygenation and ultimately ischemia and swelling of the disc. Also, a vicious cycle of ischemia, axonal swelling, microvascular compression, and further ischemia may lead to progressive nerve damage. Such progressive form is reported in 37% of NA-AION cases [9]. Nocturnal hypotension is the most important risk factor for development of NA-AION. Hence, most of the patients complain of visual loss on awakening [2]. 

Recurrent hypotensive episodes seen in an individual on dialysis for end-stage renal disease are another important risk factor for development of NA-AION [6], [7]. The incidence of symptomatic reduction in blood pressure during or immediately following dialysis ranges from 15–50% of dialysis sessions. Ghaffar et al. reported 9% of dialysis patient experience a decrease in their systolic blood pressure to below 80 mmHg during dialysis [10]. These individuals are often young with hypertension related vascular changes and also have anemia. These compounded with the frequent hypotensive episodes during dialysis makes them highly vulnerable to NA-AION, which at times may be bilateral or sequential [11], [12], [13], [14]. Our case had chronic hypertension, anemia, and end-stage renal disease requiring hemodialysis for 3 years. Occurrence of episodes of sudden loss of vision on awakening in LE a day after dialysis, pallid disc edema, delayed filling of disc in LE on FFA, no other ocular or neurologic disorder that could be responsible for disc edema, visual loss, and ambulatory blood pressure dipping to 55 mmHg confirmed the diagnosis of NA-AION secondary to hypotensive episode following hemodialysis in above patient. 

Dialysis related hypotension has a complex pathogenesis that includes insufficient vascular filling, dysfunction of the autonomous nervous system, alteration of the global diastolic ventricular function, imbalance in the vasoactive agents concentration, and feeding during the dialysis [15]. The insufficient vascular filling is caused due to depletion of volume of the interstitial space mainly by an overestimation of dry weight, low plasma osmolality with the use of low sodium concentration dialysate, decrease oncotic pressure of the capillary space or severe anemia. Dysfunction of the autonomic nervous system is due to downregulation of the adrenergic receptors. Hence, there is suboptimal compensatory cardiac and peripheral vascular resistance response to volume depletion induced by ultrafiltration. Based on these observations, it is recommended that ultrafiltration profiling, sodium-cold dialysate, and weight-based ultrafiltration are some of the measures to decrease the episodes of hypotension.

Another close differential diagnosis in patients with renal disease is uremic optic neuropathy. It is a rare entity that primarily affects individuals having highly deranged renal parameters [16]. Generally, it affects individuals with undiagnosed renal failure. Patients on maintenance dialysis are rarely affected. Visual impairment in this condition is due to direct toxic effect of dialyzable metabolites. Chronic exposure to these metabolites can lead to irreversible damage. Hence, such patients should undergo early and prompt dialysis. Knox reported significant improvement in five of six patients who underwent prompt treatment with hemodialysis and steroids [8]. Our patient was already on hemodialysis and had blood urea and serum creatinine within reasonable limits ruling out this possibility.

There is no proven therapy for NA-AION. Steroids have often being used in literature with a rationale that steroids if given during very early stages of the disease might reduce capillary permeability, leading to quicker resolution of disc edema [17]. Our patient received a course of systemic steroid but did not show any visual improvement probably because of delayed presentation. Ischemic optic neuropathy decompression trial also did not reveal any significant benefit of optic nerve decompression in NA-AION [18], [19]. A controlled clinical study of the effect of hyperbaric oxygen in NA-AION did not show any benefit with this treatment either [20]. Hence, once NA-AION is established, no treatment is effective in restoring the vision. Therefore, all care should be taken to prevent episodes of hypotension during and after dialysis. Also, single blood pressure reading may not identify patients at risk. Hence, a 24-hour ambulatory blood pressure monitoring may be done in such patients to identify early morning hypotension.

## Conclusion

Dialysis related hypotension is an important risk factor for development of NA-AION that may be recurrent or sequential. Both treating nephrologist and ophthalmologist should be aware of this potentially blinding complication. Twenty-four hour ambulatory blood pressure monitoring should be done to identify patients at risk. Care should be taken to prevent hypotensive episodes during and after dialysis.

## Notes

### Competing interests

The authors declare that they have no competing interests.

### Acknowledgement

We would like to thank Dr. Nirupama Kasturi, Asst. Professor, Department of Ophthalmology, JIPMER for her support.
